# Effect of Sudharshan Kriya Pranayama on Salivary Expression of Human Beta Defensin-2, Peroxisome Proliferator-Activated Receptor Gamma, and Nuclear Factor-Kappa B in Chronic Periodontitis

**DOI:** 10.7759/cureus.6905

**Published:** 2020-02-06

**Authors:** Ananthalakshmi Ramamoorthy, Jaideep Mahendra, Little Mahendra, Jayamathi Govindaraj, Subramaniam Samu

**Affiliations:** 1 Oral Pathology, Thai Moogambigai Dental College and Hospital, Chennai, IND; 2 Periodontics, Meenakshi Ammal Dental College and Hospital, Chennai, IND; 3 Periodontics, Maktoum Bin Hamdan Dental University College, Dubai, ARE; 4 Biochemistry, Sree Balaji Dental College and Hospital, Chennai, IND; 5 Biochemistry, Regenix Super Speciality Laboratories Research Center, Chennai, IND

**Keywords:** periodontitis, hbd-2, ppar-γ, nf-κb, yoga, sudharshan kriya pranayama

## Abstract

Introduction

Sudharshan kriya pranayama (SKP) is a form of yoga that reduces inflammation and contributes to general health. Very few prior studies have examined the role of pranayama on oral health. We evaluated the clinical status and inflammatory biomarkers in patients with chronic periodontitis before and after SKP.

Materials and methods

Ninety male subjects were considered for the study and divided into three groups: subjects with a healthy periodontium (Group1), subjects with chronic gingivitis (Group2), and subjects with chronic periodontitis (Group3). The clinical parameters included plaque index (PI), gingival index (GI), probing pocket depth (PPD), clinical attachment level (CAL), and salivary markers human beta-defensin-2(HBD-2), peroxisome proliferator-activated receptor gamma (PPAR-γ), and nuclear factor-kappa B (NF-κB). These parameters and markers were evaluated before and after 90 days of SKP. The data obtained were statistically evaluated by McNemar's test, paired sample t-test, and one-way analysis of variance.

Results

There was a significant improvement in PI in all three groups. GI showed an improvement in Groups 2 and 3. PPD and CAL also showed an improvement in Group 3. HBD-2 and NF-κB decreased with SKP, whereas PPAR-γ expression increased after the intervention. In Groups 2 and 3 with the decrease in GI, there was a corresponding decrease in HBD-2. In Group 3 with an improvement in PPD and CAL, there was an improvement in PPAR-γ expression.

Conclusions

The results show that SKP can significantly decrease periodontal inflammation and improve periodontal status. It also effectively improves the expression of PPAR-γ, thereby decreasing salivary levels of HBD-2 and NF-κB, respectively. Based on our results, pranayama can be an effective adjunct in maintaining oral health.

## Introduction

Periodontitis is the most common oral inflammation [1]. Various reviews on the prevalence of periodontal disease identified a high prevalence among Indian adults (95%), specifically in the economically weaker population [2-3]. Due to the current socioeconomic status of developing countries like India, health care is expensive and cannot be afforded by everyone. In such a scenario, 5,000-year-old complementary and alternative medicine practices such as yoga have proven to be a cost-effective aid in the physical, psychological, and social well-being of individuals, thereby increasing innate immunity. Yoga has been demonstrated as a curative measure for the modifiable and non-modifiable risk factors of periodontitis [4]. Numerous researchers have proven the positive effect of Sudharshankriya on various systemic and psychological diseases and shown a favorable effect on inflammation [5].

Defensins are secreted by the oral mucosal surfaces. They are a marker with high sensitivity and specificity in saliva [6]. Human beta-defensin-2(HBD-2) is a cationic peptide influenced by the severity of the periodontal disease. Nuclear factor-kappa B (NF-κB) induces an inflammatory response in periodontal structures, whereas peroxisome proliferator-activated receptor gamma(PPAR-γ) has an anti-inflammatory effect by down-regulating NF-κB [7]. Though prior research has been done regarding pranayama and general health, there is little data on the role of pranayama on oral inflammation. Newer treatment strategies for periodontal disease are focusing on naturopathy and its influence on biochemical links to inflammation [8].

We analyzed the effect of Sudharshan Kriya pranayama (SKP) on the levels of salivary HBD-2, NF-κB, PPAR-γ, and periodontal parameters of subjects with gingival and periodontal disease. We hypothesized that via the practice of SKP, levels of proinflammatory markers such as NF-κB and anti-inflammatory markers such as HBD-2 and PPAR-γ could be regulated, thereby preventing further progression of periodontal disease and maintaining overall oral health.

## Materials and methods

In this study, 110 men between the ages of 18 and 35 years with a minimum of 24 teeth present were recruited from the Art of Living (AOL) Center, Chennai, India. Of these, nine did not agree to be the part of the study, three were hypertensive, two had diabetes, five were smokers, and one subject had periodontal therapy four months before, and therefore, they were excluded from the study. A total of 90 men were selected for the study; the omission of women is a limitation of the study design as discussed below. After the periodontal screening, the participants were divided into three groups: Group 1 composed of 30 systemically and periodontally healthy subjects; Group 2 composed of 30 subjects with chronic gingivitis; and Group 3 composed of 30 subjects with chronic periodontitis. Institutional ethical clearance was provided by the Meenakshi Academy of Higher Education and Research (MAHER) (MAHER-MU-002-IEC/2016) following the Declaration of Helsinki. The study was conducted after obtaining informed written consent from each participant.

Clinical data collection

The periodontal evaluation covered plaque index (PI), gingival index (GI), probing pocket depth (PPD), and clinical attachment level (CAL) [9]. The PI was recorded with an interpretation of 0=no plaque, 1=film of plaque, 2=moderate accumulation of deposits, and 3=abundance of deposits [8]. The GI was done with an interpretation of 0= no inflammation, 0.1 to 1= mild inflammation, 1.1 to 2= moderate inflammation, and 2.1 to 3= severe inflammation. The PPD was measured from the gingival margin to the base of the periodontal pocket [10]. The CAL was measured as the distance from the cementoenamel junction to the base of the periodontal pocket [10].

Sample collection

A total of 5 ml of unstimulated saliva sample was collected at baseline and after the 90-day practice of SKP. Saliva samples were stored at -50°C and preserved until the end of the study. The samples were thawed before processing. Levels of HBD-2 were estimated by enzyme-linked immunosorbent assay (Qayee bio-technology Cat.No. 100-250-BD2) while PPAR-γ, and NF-κB were estimated by a polymerase chain reaction.

Intervention

SKP was used as an intervention. The session was held from 6 AM to 7 AM. A Certified yoga teacher of the AOL taught and monitored the practice for 90 days. The kriya started with three stages of ujjayipranayama (victorious breath), followed by three cycles of bhastrika pranayama (bellow breath), and then with Om chanting three times. The last stage of SKP was with normal breathing in slow, medium, and fast cycles followed by rest [11].

Statistical analysis

Statistical analysis was done with SPSS for Windows, Version 16.0 (SPSS Inc., Chicago). A McNemar's test was done to compare clinical parameters before and after the intervention, a paired sample t-test was used to compare the pre- and post-intervention mean values of biologic parameters, and one-way analysis of variance was used to compare the parameters between groups at the end of the study. P<.001 was considered statistically significant.

## Results

The results showed a statistical improvement in PI in all three groups after 90 days of SKP (P<.001) (Table 1, Table 2, Table 3). GI also showed significant improvement in Groups 2 and 3 after SKP (P<.001; Table 2, Table 3). Group 3 had significant improvements in mean PPD and CAL when compared to baseline after SKP (Table 3).

**Table 1:** Comparison of clinical and biological parameters before and after Sudharshan Kriya pranayama among the healthy group (Group 1) CAL, clinical attachment level; GI, gingival index; HBD-2, human beta-defensin-2; NF-κB, nuclear factor-kappa B; PI, plaque index; PPAR-γ, peroxisome proliferator-activated receptor gamma; PPD, probing pocket depth.

		N	Mean	Standard Deviation	P	95% Confidence Interval for Mean		Minimum	Maximum
						Lower Bound	Upper Bound		
PI	PRE	30	.9667	.51350	.001	.7749	1.1584	.00	1.80
	POST	30	.4533	.33190		.3294	.5773	.00	1.20
	Total	60	.7100	.50075		.5806	.8394	.00	1.80
GI	PRE	30	.0000	.00000	0	.0000	.0000	.00	.00
	POST	30	.0000	.00000		.0000	.0000	.00	.00
	Total	60	.0000	.00000		.0000	.0000	.00	.00
PPD	PRE	30	2.2000	.40684	.009	2.0481	2.3519	2.00	3.00
	POST	30	2.0000	.00000		2.0000	2.0000	2.00	2.00
	Total	60	2.1000	.30253		2.0218	2.1782	2.00	3.00
CAL	PRE	30	2.2000	.40684	.009	2.0481	2.3519	2.00	3.00
	POST	30	2.0000	.00000		2.0000	2.0000	2.00	2.00
	Total	60	2.1000	.30253		2.0218	2.1782	2.00	3.00
HBD-2	PRE	30	91.6000	5.82859	.003	89.4236	93.7764	79.00	99.00
	POST	30	96.2000	5.80368		94.0329	98.3671	88.00	105.00
	Total	60	93.9000	6.21562		92.2943	95.5057	79.00	105.00
NF-κB	PRE	30	-.2630	.19988	.001	-.3376	-.1884	-.59	.09
	POST	30	-.7720	.29767		-.8832	-.6608	-1.14	-.16
	Total	60	-.5175	.35925		-.6103	-.4247	-1.14	.09
PPAR-γ	PRE	30	1.4130	.49705	.001	1.2274	1.5986	.43	1.99
	POST	30	1.7533	.24528		1.6617	1.8449	1.18	2.02
	Total	60	1.5832	.42480	.001	1.4734	1.6929	.43	2.02

**Table 2:** Comparison of clinical and biological parameters before and after Sudharshan Kriya pranayama among the chronic gingivitis group (Group 2) CAL, clinical attachment level; GI, gingival index; HBD-2, human beta-defensin-2; NF-κB, nuclear factor-kappa B; PI, plaque index; PPAR-γ, peroxisome proliferator-activated receptor gamma; PPD, probing pocket depth.

		N	Mean	Standard Deviation	P	95% Confidence Interval for Mean		Minimum	Maximum
						Lower Bound	Upper Bound		
PI	PRE	30	1.8600	.56116	< .001	1.6505	2.0695	1.00	2.90
	POST	30	1.2900	.65566		1.0452	1.5348	.00	3.00
	Total	60	1.5750	.66984		1.4020	1.7480	.00	3.00
GI	PRE	30	1.8700	.52467	< .001	1.6741	2.0659	1.00	2.80
	POST	30	1.0200	.56471		.8091	1.2309	.20	2.50
	Total	60	1.4450	.68974		1.2668	1.6232	.20	2.80
PPD	PRE	30	2.2333	.43018	.004	2.0727	2.3940	2.00	3.00
	POST	30	2.0000	.00000		2.0000	2.0000	2.00	2.00
	Total	60	2.1167	.32373		2.0330	2.2003	2.00	3.00
CAL	PRE	30	2.0000	.00000	0	2.0000	2.0000	2.00	2.00
	POST	30	2.0000	.00000		2.0000	2.0000	2.00	2.00
	Total	60	2.0000	.00000		2.0000	2.0000	2.00	2.00
HBD-2	PRE	30	110.10	7.89740	< .001	107.1511	113.0489	99.00	125.00
	POST	30	98.3000	5.93151		96.0851	100.5149	89.00	109.00
	Total	60	104.20	9.12958		101.8416	106.5584	89.00	125.00
NF-κB	PRE	30	.4550	.44481	.001	.2889	.6211	-.12	1.18
	POST	30	-.4820	.33120		-.6057	-.3583	-.92	-.05
	Total	60	-.0135	.61187		-.1716	.1446	-.92	1.18
PPAR-γ	PRE	30	.2970	.88469	.001	-.0333	.6273	-.85	1.43
	POST	30	.9513	.52997		.7534	1.1492	.05	1.72
	Total	60	.6242	.79474		.4189	.8295	-.85	1.72

**Table 3:** Comparison of clinical and biological parameters before and after Sudharshan Kriya pranayama among the chronic periodontitis group (Group 3) CAL, clinical attachment level; GI, gingival index; HBD-2, human beta-defensin-2; NF-κB, nuclear factor-kappa B; PI, plaque index; PPAR-γ, peroxisome proliferator-activated receptor gamma; PPD, probing pocket depth.

		N	Mean	Standard Deviation	P	95% Confidence Interval for Mean		Minimum	Maximum	
						Lower Bound	Upper Bound			
PI	PRE	30	2.2233	.45538	< .001	2.0533	2.3934	.80	2.90	
	POST	30	1.6033	.57023		1.3904	1.8163	.50	2.70	
	Total	60	1.9133	.59957		1.7584	2.0682	.50	2.90	
GI	PRE	30	2.2400	.40565	< .001	2.0885	2.3915	1.50	2.90	
	POST	30	1.5967	.48172		1.4168	1.7765	.50	2.10	
	Total	60	1.9183	.54787		1.7768	2.0599	.50	2.90	
PPD	PRE	30	5.8600	.58933	< .001	5.6399	6.0801	4.80	6.80	
	POST	30	5.0733	.61135		4.8451	5.3016	3.80	6.20	
	Total	60	5.4667	.71537		5.2819	5.6515	3.80	6.80	
CAL	PRE	30	5.4000	.51862	< .001	5.2063	5.5937	4.60	6.40	
	POST	30	4.6133	.60386		4.3878	4.8388	3.50	6.10	
	Total	60	5.0067	.68467		4.8298	5.1835	3.50	6.40	
HBD-2	PRE	30	159.30	12.06920	< .001	154.7933	163.8067	143.00	180.00
	POST	30	131.00	14.04181		125.7567	136.2433	109.00	152.00	
	Total	60	145.15	19.29066		140.1667	150.1333	109.00	180.00	
NF-κB	PRE	30	2.3180	.27438	< .001	2.2155	2.4205	1.99	2.74
	POST	30	-.1217	.34264		-.2496	.0063	-.58	.59	
	Total	60	1.0982	1.26804		.7706	1.4257	-.58	2.74	
PPAR-γ	PRE	30	-1.2710	.75110	< .001	-1.5515	-.9905	-1.99	.26
	POST	30	1.7433	.32038		1.6237	1.8630	1.26	2.14	
	Total	60	.2362	1.62413		-.1834	.6557	-1.99	2.14	

Levels of HBD-2 and NF-κB expression were significantly reduced after 90 days of SKP in Groups 2 and 3 (Table2, Table 3). Mean HBD-2 levels decreased significantly, correlating with inflammation in Groups 2 and 3, after the intervention (P<.001). Alternatively, the mean expression of PPAR-γ increased significantly from baseline after the SKP intervention in Group 3 (P<.001), whereas the expression of NF-κB decreased significantly after 90 days of SKP (P<.001; Table 2, Table 3).

## Discussion

Chronic periodontal disease is a form of oral inflammation that affects the gingival and periodontal tissues, ultimately causing loss of alveolar bone. Periodontal diseases such as chronic periodontitis can affect the progression of systemic diseases, thereby affecting the overall health of the individual [12]. Complications from periodontal inflammation and its causative pathogens are not restricted to the oral cavity. These infections can be related to major systemic diseases such as subacute bacterial endocarditis, rheumatoid arthritis, ocular disease, skin disorders, gastrointestinal, renal, and even pregnancy complications like low birth weight, preterm birth, and pre-eclampsia [13]. SKP is a form of yoga that has promising effects on psychological stress, thus boosting innate immunity against oral inflammation.

HBD-2, an innate immunity marker, is antimicrobial and has been proven to increase after yogic stretching. HBD-2 has a potent role in the pathogenesis of caries, periodontal disease, and oral squamous cell carcinoma [14]. NF-κB is a protein complex that regulates DNA transcription, the production of cytokines, and cell survival. NF-κB is found in almost all cell types and is involved in cellular responses to stimuli such as stress. It has an important role in the onset of periodontal inflammation and inhibits osteoblastic bone formation [15]. Similarly, PPAR-γ is a transcription factor and an anti-inflammatory molecule considered to improve immunity. SKP decreases inflammation through the selective effect on regulatory hormones via an alteration in the hypothalamic-pituitary-adrenal and sympathetic-parasympathetic axes [16]. Because there are no data demonstrating the impact of pranayama on levels of HBD-2, PPAR-γ, and NF-κB in oral inflammation, we examined the levels of salivary HBD-2, NF-κB, and PPAR-γ in subjects with the periodontal disease using SKP as an intervention.

In our study, after the 90-day practice of SKP, the mean PI score was significantly reduced, indicating an improvement in the oral hygiene of all participants in all groups. These results were in accordance with those of Ananthalakshmi et al. and Farsi et al. [10,17]. Farsi stated that the plaque index increases with decreases in salivary flow. SKP increased the flow rate of saliva and thereby maintained oral health. This is in accordance with Eda et al., who stated that yogasana would increase the rate of saliva flow, a natural oral cleanser [18]. As plaque is one factor that directly determines the severity of periodontitis, improving oral hygiene will decrease the incidence of periodontitis. In our study, SKP as an intervention reduced PI and thereby improved oral health.

Gingival inflammation is a stepping stone for periodontal inflammation; its treatment prevents the onset of periodontal disease. In our study, mean inflammatory scores for GI were reduced significantly in Groups 2 and 3 after SKP. Gingivitis is a pathologic response to microbial byproducts, leading to proinflammatory cytokines such as interleukin (IL)-1, IL-6, and IL-8, tumor necrosis factor (TNF), and the like, that leads to the destruction of the periodontium [19]. SKP increases antioxidant levels, thereby decreasing levels of proinflammatory cytokines and reactive oxygen species, thus breaking the inflammatory cascade. Sharma et al. also noted an increase in antioxidant levels after SKP [20]. This was in accordance with Mahendra et al., who found a significant decrease in bleeding index in subjects after SKP intervention [7].

Mean PPD and CAL on the 90th day of SKP practice were found to have improved from baseline in chronic periodontitis patients (Group 3), indicating an improvement in pocket depth and clinical attachment level. Gingival fibroblasts play a key role in the healing of periodontium, thereby improving PPD and CAL. Apoptosis of these fibroblasts is reduced with SKP, thereby strengthening the periodontium. This was in accordance with Mahendra et al. [7] and Su Qu et al. [21]. In the Su Qu study of gene expression profiling after SKP intervention, he observed that there was an increase in the expression of genes that prevented apoptosis (such as COX2, BCL2) [21].

HBD-2 is a cationic peptide upregulated by the byproducts of periodontitis pathogens. The virulence of organisms like *Aggregatibacter actinomycetemcomitans, Porphyromonas gingivalis,* and *Fusobacterium nucleatum* influences periodontal inflammation. Hosokawa et al. estimated a higher concentration of HBD-2 in the chronic periodontitis group followed by the gingivitis and healthy groups [22]. Similarly, in our study, HBD-2 was found to be higher in the chronic periodontitis group than the gingivitis and healthy groups both at baseline and after the intervention. These interpretations are in accordance with Kuula et al. and Periere et al [23-24]. The anti-inflammatory effects of SKP down-regulated HBD-2 and improved periodontal status in our study group.

In periodontal diseases, NF-κB differentiates and progresses osteoclastic activity, whereas PPAR-γ is an anti-inflammatory nuclear hormone receptor that modulates NF-κB signaling. The release of PPAR-γ counteracts the effects of NF-κB [7]. Our results showed the down-regulation of NF-κB and the upregulation of PPAR-γ in Group 3. These results were in agreement with a study conducted using SKP as an intervention by Mahendra et al. [7]. Brand et al. also stated the role of NF-κB in systemic inflammation, such as atherosclerosis, which was effectively treated with SKP [25-26].

Yogic practice is considered superior to traditional exercise as results obtained after high-intensity workouts are quantitative at the muscular level when compared with the qualitative output of yoga practice that leads to the energization of the chakras (body energy centers) [27]. Our study had two important limitations. We neither did explore periodontal pathogens nor did we conduct a clinical evaluation of periodontitis in women with SKP intervention. Future studies of periodontal microbiology should compare periodontal inflammatory markers after SKP intervention and include female patients, thereby elucidating the role of SKP on these factors.

## Conclusions

SKP effectively decreased periodontal inflammation, HBD-2 levels, and NF-κB expression, thereby maintaining healthy periodontium. In addition, SKP increased the expression of PPAR-γ, which modulates NF-κB, thereby improving the host immune response. In the future, SKP can be considered as an important adjunct to an integrated inflammatory treatment modality.
